# Potential group B *Streptococcus* interspecies transmission between cattle and people in Colombian dairy farms

**DOI:** 10.1038/s41598-019-50225-w

**Published:** 2019-10-01

**Authors:** Claudia G. Cobo-Angel, Ana S. Jaramillo-Jaramillo, Monica Palacio-Aguilera, Liliana Jurado-Vargas, Edwin A. Calvo-Villegas, Diego A. Ospina-Loaiza, Juan C. Rodriguez-Lecompte, Javier Sanchez, Ruth Zadoks, Alejandro Ceballos-Marquez

**Affiliations:** 1 Research Group in Milk Quality and Veterinary Epidemiology, Faculty of Agricultural Sciences, Universidad de Caldas, Manizales, Colombia; 2 Faculty of Health Sciences, Universidad de Caldas, Manizales, Colombia; 3 Atlantic Veterinary College, University of Prince Edward Island, Charlottetown, Prince Edward Island Canada; 4 Institute of Biodiversity, Animal Health and Comparative Medicine, College of Medical, Veterinary and Life Sciences, University of Glasgow, Glasgow, UK; 5 Sydney School of Veterinary Science, University of Sydney, Camden, NSW Australia

**Keywords:** Applied microbiology, Infectious-disease diagnostics, Policy and public health in microbiology, Bacterial infection, Neonatal sepsis

## Abstract

Group B *Streptococcus* (GBS), is a leading cause of neonatal death and an emerging pathogen in adults. Additionally, GBS is a bovine pathogen causing intramammary infections. The likelihood of GBS interspecies transmission is largely unknown. We explored the potential transmission of GBS between cattle and people on dairy farms in Colombia and compared the antimicrobial resistance (AMR) profiles of isolates from both host species. Across 33 farms, throat swabs and rectal swabs were collected from 191 people, and rectal swabs and composite milk samples from 2092 cattle, yielding 60 human isolates and 301 bovine isolates. The majority (64%) of isolates belonged to shared sequence types (ST). Sequence type (ST) 1 was the most common strain in both host species, suggesting that interspecies transmission may be possible. Two members of the bovine-specific clonal complex 61/67 were detected in human samples (ST718 and ST1175), providing evidence for the lack of genuine species barriers. Apparent prevalence of penicillin resistance was surprisingly high in human and bovine isolates. Further investigation of this phenomenon is needed and could lead to modification of standard testing and treatment recommendations in human and veterinary medicine.

## Introduction

*Streptococcus agalactiae* or Group B *Streptococcus* (GBS) is an important etiologic agent in a wide variety of human infections. GBS is often carried asymptomatically by healthy adults, ranging from 20 to 40% in developed countries^1–4^. In South America, the reported prevalence of GBS colonization in pregnant women ranges from 6% to 26%, depending on country and culture methods^5–8^. In pregnant women, GBS may reach the amniotic fluid and fetal membranes, and cause fetal deaths^9^. Furthermore, GBS is a severe neonatal infections such as sepsis, meningitis and pneumonia^10^. Despite the fact that GBS colonization may lead to neonatal infection, the predominant strains associated with carriage, notably sequence type (ST)1, ST19 and ST23, differ from the major neonatal clade, ST17^11^.

In adults, GBS can cause bacteremia, respiratory infections, urinary tract infections, joint and bone infections, endocarditis or meningitis, and skin and soft tissues infections, particularly in elderly and immunocompromised patients^11,12^. Surveillance in the United States of America (USA) suggests that GBS infection is an emerging disease in nonpregnant adults, where ST1 and ST196 have been recognized as emerging clades^11^.

Group B *Streptococcus* is also a bovine pathogen causing intramammary infections leading to mastitis^13^. The prevalence of GBS in dairy herds varies from less than 10% in Canada^14,15^ and northern European countries^16–18^, to approximately 50% in South America^19,20^, and more than 90% in China^21^. Based on studies in epidemiologically unrelated populations, it has been suggested that bovine and human strains are largely distinct populations and interspecies transmission is unlikely^22,23^. There are no studies on this topic from emerging economies where the epidemiology of GBS may be different from high income countries with advanced animal health infrastructure^24,25^. To elucidate the molecular epidemiology of GBS, typing methods must have high typeability and discriminatory power. Serotyping of GBS, which is widely used in human medicine, only identifies 10 serotypes so it has very limited discriminatory ability. Moreover, many bovine isolates are not typeable by means of serotyping^26^, which is possibly due to pseudogenization of the capsular operon in bovine isolates^27^. Even among human isolates, more than 12% may be non-typeable based on serotyping^8^. For those reasons, multilocus sequence typing (MLST) is a more suitable typing method for epidemiological studies, particularly those involving cattle. In a European study of contemporaneous sympatric isolates from humans and cattle, five shared STs comprised more than half of the GBS isolates in the study^28^.

Comparison of isolates that were collected in the same countries in the same years was an improvement over comparisons based on epidemiologically unrelated isolates, but there was no known contact between people and cattle. In a study of 68 farming families and their cattle in the United States, a shared ST was detected in a cow’s feces and in stool of a couple living on the farm^29^. These findings support the possibility of on-farm interspecies transmission. This phenomenon has not been studied in countries with high GBS prevalence in the cattle population.

In human and bovine medicine, the treatment of choice for GBS infection consists of beta-lactam antibiotics, specifically penicillin^13–30^. The drugs of second choice, particularly in people with penicillin allergies, members of the Macrolide-Lincosamide-Streptogramin group such as the macrolide erythromycin and the lincosamide clindamycin^31^. Decreased susceptibility of human GBS strains has been reported for penicillin^32^, erythromycin and clindamycin^33,34^. A study in Colombia, showed that 20% of cows treated with beta-lactam antibiotics remained GBS positive after use in accordance with the pharmaceutical company’s recommendations^35^. One potential explanation for this observation is the emergence of antimicrobial resistance (AMR) in bovine GBS. This has been described in China and may be the evolutionary consequence of routine use of antimicrobials for infection control^36^. Interspecies transmission of resistant GBS is thought to occur based on tetracycline resistance profiles of human and bovine GBS isolates^26^. The emergence of penicillin resistance in South American dairy cattle would aggravate concerns about interspecies transmission of GBS.

We investigated the potential for on-farm transmission of GBS between cows and people on dairy farms using MLST and AMR testing to understand possible public health risks of dairy farming in areas with high GBS prevalence in cattle, and to explore the need to update current GBS mastitis control recommendations.

## Results

### Group B *Streptococcus* colonization

Of 189 people from 33 farms tested by rectal and throat swab, 15 (8%) were positive by both swabs, 20 (11%) by rectal swab only and 10 (5%) by throat swab only. Human carriers were detected on 25 (76%) of 33 farms. The frequency of GBS isolation was significantly different between throat and rectum (P < 0.001) (Table 1). Detection of GBS at both sites was almost 3x as many as expected under the assumption of independence of rectal and pharyngeal colonization. GBS was found in cows from 28 (85%) of 33 herds. Detection was more common in milk than rectal samples (P < 0.001), with 261 (12.5%) GBS positives among 2092 cows tested using milk and 40 (2.1%) GBS positives among 1992 cows tested using rectal swabs (Table 1). On 22 (66.6%) farms, GBS was isolated from both farmers and cattle, which is almost equal to the proportion expected based on independence of GBS presence in humans in cattle. Isolation from environmental samples was rare (Table 1; breakdown by herd in Supplementary Table S1).

**Table 1:** Farm level prevalence and sample level prevalence of group B *Streptococcus* (GBS) on 33 dairy farms.

Source of isolate		GBS positive herds n (%)	GBS isolates n (%)	GBS positive samples per farm
Farmers	Throat (n = 191)	15 (45.5)	25 (13.2)^a^	0–4
	Rectum (n = 189)	22 (66.7)	35 (18.5)^b^	0–4
Cows	Mammary gland (n = 2092)	27 (81.8)	261 (12.5)^a^	0–35
	Rectum (n = 1922)	11 (33.3)	40 (2.1)^b^	0–16
Cows’ Environment	Feeders (n = 128)	3 (9.1)	3 (2.3)	0–1
	Drinking water (n = 47)	2 (6.1)	2 (4.3)	0–1

Different letters indicate significant differences at P < 0.05 in the frequency of GBS isolation between body sites within host species, according to Pearson χ^2^ test.

### MLST results

**Figure 1:**
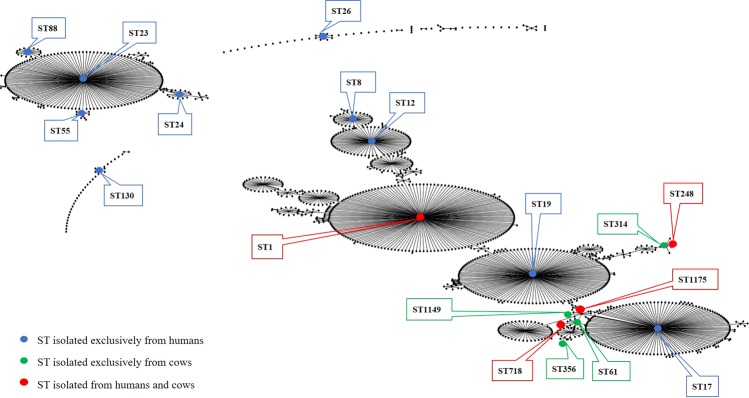
Population snapshot showing all known sequence types (ST) of Group B *Streptococcus* (GBS) with STs found in the current study shown in color.

Among a grand total of 366 GBS isolates from human (n = 60), bovine (n = 301) and environmental (n = 5) samples, 18 STs belonging to eight CCs were detected (Fig. 1; Table 2). Most STs were predominantly or exclusively found in a single host species. The exception was ST1, which was common in both sample types from people and cattle (Table 2; details in Supplementary Table S1). ST248 and ST1175 were also found across both host species (3 to 12% per body site), whilst ST718 was the third most common type in cattle but very rare in people (Table 2).

**Table 2:** Number (n) and proportion (%) of samples and group B *Streptococcus* by sample origin, clonal complex (CC) and sequence type (ST).

CC	ST	Human		Bovine		Environmental (n = 5) n (%)
		Throat (n = 25) n (%)	Rectum (n = 35) n (%)	Milk (n = 261) n (%)	Rectum (n = 40) n (%)	
1	1	7 (28)	10 (29)	106 (41)	9 (23)	2 (40)
12	8		1 (3)			
	12		1 (3)			
	130	2 (8)	3 (9)			
17	17	3 (12)	1 (3)			
19	19		1 (3)			
23	23	6 (24)	3 (9)			
	24		6 (17)			
	55	1 (4)	1 (3)			
	88	2 (8)	3 (9)			
26	26		3 (9)			
61/67	61			15 (6)		
	356			55 (21)	10 (25)	3 (60)
	718	1 (4)		55 (21)	2 (5)	
	1149			4 (2)	16 (40)	
	1175		2 (6)	9 (3)		
103	248	3 (12)		12 (5)	3 (8)	
	314			5 (2)		

**Figure 2:**
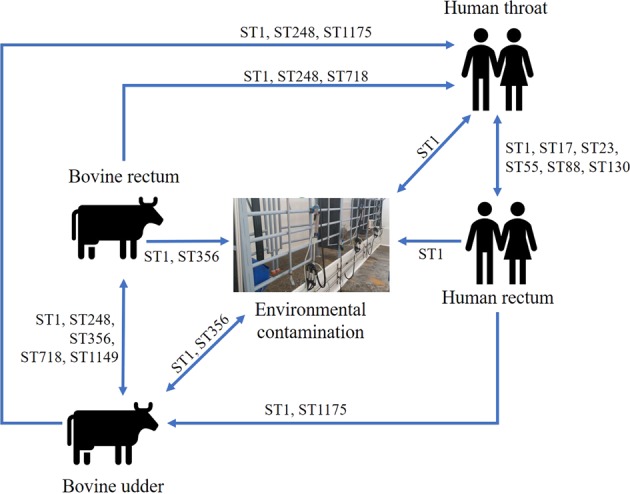
Hypothetical Group B *Streptococcus* transmission routes between people, cows, and the dairy farm environment, based on sequence types (ST) that were found across multiple sample types on 33 dairy farms in Colombia, with arrows showing potential directions of transmission.

Of 366 isolates obtained in this study, 234 (64%) belonged to shared STs, including 23 (38%) human isolates and 196 (65%) bovine isolates. Shared STs were detected on 7 of 22 (31.8%) farms where both host species tested positive for GBS. ST1 was the shared type on 6 farms and ST718 on one farm. On two farms, ST1 was found in the cows’ environment and a throat swab (Supplementary Table S1). Hypothetical transmission routes between niches, based on shared STs are summarized in Fig. 2.

### Antimicrobial resistance

Of 366 isolates, 269 (73.5%) were viable after prolonged storage and available for phenotypic and genotypic antimicrobial susceptibility testing. This included 33 (55%) of 60 human isolates representing 12 of 14 STs identified among human GBS, and 236 (78.4%) of 301 bovine isolates, including all STs identified among bovine GBS in this study (Supplementary Table S2). The apparent prevalence of resistance depended on the choice of interpretation criteria (Supplementary Table S3), particularly for penicillin resistance in bovine isolates. Based on CLSI criteria, only 46% of bovine isolates were classed as penicillin susceptible compared to 76% based on EUCAST criteria. Regardless of host species and the source of interpretation criteria, most isolates were susceptible to ampicillin and erythromycin but resistant to tetracycline (Table 3).

**Table 3:**
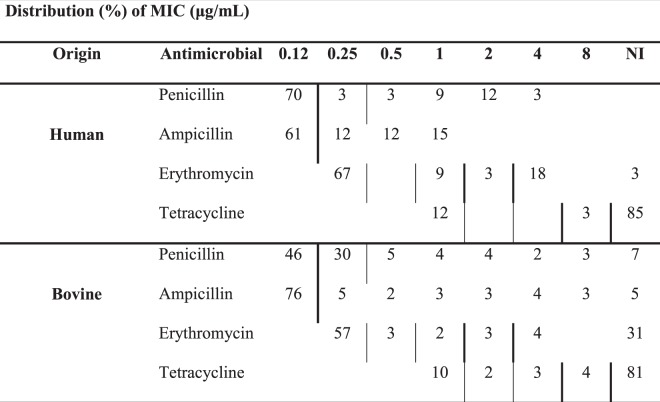
Distribution of minimum inhibitory concentration (MIC) among Group B *Streptococcus* isolates form people (n = 33) and cattle (236) on dairy farms.

Thick vertical lines indicate breakpoints set by the Clinical and Laboratory Standards Institute, 2018. Thin vertical lines indicate breakpoints set by the European Committee on Antimicrobial Susceptibility Testing, 2019. NI = no inhibition.

Non-susceptibility to tetracycline (based on CLSI criteria) was significantly associated with detection of *tet*(M) (P = 0.001) and *tetO* (P = 0.020), but not *tet*(K), which was detected in only 5 isolates. Tetracycline resistance encoded by *tet*(M) was significantly more common among bovine isolates than among human isolates (Table 4). It was overrepresented in ST1 compared to non-ST1 and in ST718 compared to non-ST718 (*P* < 0.001 for both comparisons; Supplementary Table S3). Non-susceptibility to erythromycin (based on CLSI criteria) was significantly associated with detection of *ermB* (*P* < 0.001) but not *ermA* (*P* = 0.27). The efflux pump gene *mefA* was only detected in two human isolates. The prevalence of *ermA* or *ermB* was not significantly different between human and bovine isolates (Table 4). Resistance determinant *ermA* was overrepresented in ST1 compared to non-ST1 (*P* = 0.02) whilst *ermB* was overrepresented in ST1 compared to non-ST1, in ST356 compared to non-ST356 and in ST718 compared to non-ST718 (*P* < 0.001, *P* < 0.001 and *P* = 0.004, respectively; Supplementary Table S3).

**Table 4:** Prevalence of resistance genes in Group B *Streptococcus* (GBS) isolated from people (33 isolates) and cattle (236 isolates) on dairy farms in Colombia and significance of the association with host species.

Antimicrobial class	Resistance gene	GBS source		P-value
		Human n (%)	Bovine n (%)	
Macrolides	*ermA*	8 (24)	33 (14)	0.12
	*ermB*	13 (39)	125 (53)	0.07
	*mefA*	2 (6)	0 (0)	Not tested
Tetracyclines	*tet*(K)	3 (9)	2 (1)	0.001
	*tet*(M)	18 (55)	174 (74)	0.02
	*tet*(O)	6 (18)	32 (14)	0.24

## Discussion

Here, we demonstrate the existence of GBS strains (based on MLST) that are shared between farmers and cattle within dairy farms, including the highly prevalent ST1. Previous studies have suggested the possibility of zoonotic or anthroponotic transmission based on characterization of contemporaneous and sympatric isolates from humans and bovines without known epidemiological connections^26–28^. All human participants in our study lived on farms with dairy cattle, and about the half of them participated directly in the milking process. Those that did not participate in milking were likely to be in contact with cattle, cattle manure, farm run-off, or milk, which is often consumed raw in Colombia, particularly on dairy farms^37^, in contrast to the situation in high-income countries were most work on GBS epidemiology has been conducted. Based on our MLST results and potential transmission mechanisms^28^, interspecies transmission may occur in both directions. For example, consumption of raw milk may explain occurrence of shared STs between bovine milk samples and human throat swabs, whereby GBS in milk may originate from intramammary infection (Fig. 2, ST1, ST248 and ST1175) or from fecal contamination of milk with the bovine rectum as the ultimate source (Fig. 2, ST1, ST248 and ST718). Human sputum or feces may contaminate the environment (Fig. 2, ST1) and rectal GBS from humans could be transferred to the bovine mammary gland via unwashed hands, especially when gloves are not used during milking (Fig. 2, ST1175). Demonstrating the occurrence of such transmission events based on longitudinal studies is challenging, but transmission from animals to humans via raw food^38^ and from humans to animals via excreta has been described in outbreak situations involving fish^39^.

Sequence type 1 was detected in every sample type and it was the dominant strain in both host species, in agreement with previous studies^28,29^. ST1 is emerging as a leading cause of invasive disease in non-pregnant adults, which is attributed host adaptation through a series of small and cumulative genetic events^40^. The ability of bacteria to adapt to different host species and to acquire additional virulence genes or resistance genes in different functional environments poses a major threat to public health and food safety^41^. It may lead to repeated host switching, a phenomenon that has been demonstrated for *Staphylococcus aureus*. Like GBS, this *S. aureus* colonizes and infects humans cattle and other host species, and is a major cause of bovine mastitis^41^. It is conceivable that GBS could also evolve to switch between human and animal hosts, acquiring accessory genome content from different niches in the process.

In contrast to ST1, members of clonal complex (CC) 61/67 are considered to represent a host-adapted clade of GBS. This was the first CC of GBS to be described in cattle, and it is still the predominant cattle clade in bovines in parts of Europe^27–42^. Adaptation to cattle involves acquisition of bovine-specific virulence factors, e.g. the lactose operon^43^ as well as loss of human-specific virulence factors e.g. capsule^27^. In our study, members of CC61/67 were also isolated from human. We repeated GBS isolation and MLST analysis to rule out laboratory errors and confirm this unexpected finding. To our knowledge, we report CC61/67 carriage in humans for the first time here. Meanwhile, CC61/67 has also been isolated from human clinical samples from patients with vaginitis and urethritis in Asia^44^. Their emergence as human pathogen was associated acquisition of the CC17-specific type C gbs2018 gene^44^. It underscores the risk of ongoing evolution and expansion of the host range of GBS clades. Likewise, CC103, is a common cause of mastitis in dairy cattle in places as far apart as northern Europe^28–45^, China^46^, and Colombia^25^, whereas it has only rarely been reported in humans^47^. In the current study, CC103 member ST248 was the second most common strain shared by people and cattle. It has also been reported as a human strain in China^44^. The examples given here show the lack of a genuine species barriers, at least at ST level. Simple interventions aimed at improved hygiene during milking harvesting and consumption could reduce the risk of transmission of GBS between cattle and people. For instance, avoid raw milk consumption could interrupt the transmission from cattle to people. Improved sanitation and hand hygiene or use of gloves during milking could prevent transmission from people to cattle.

The Centers for Disease Control and prevention (CDC) in the USA describe GBS as completely susceptible to beta lactams antibiotics and CLSI reports that GBS isolates that are non-susceptible to beta lactams are extremely rare^48^. However, penicillin resistance in GBS has been reported from Asia, including among hospital isolates from humans in Japan^49^, and isolates from tilapia^50^ and dairy cattle in China^36^. Results from our study suggest that penicillin resistance may also be emerging in South America. Interpretation of our data is somewhat hampered by the fact that we used standard methods for veterinary microbiology rather than the CLSI or EUCAST protocols. In addition, there are no guidelines for the interpretation of penicillin MIC data for cattle. Despite those caveats, our results give reason for concern. Further investigation of this phenomenon is needed, especially considering that penicillin is currently the treatment of choice in both host species. No evidence was found of penicillin resistance in human carriage isolates in Japan^51^, suggesting that antimicrobial use in clinical settings, agriculture and aquaculture may exert the selective pressure needed to provide penicillin resistant GBS with a survival advantage. This emphasizes the need to use antimicrobials judiciously, and to focus on prevention of GBS transmission in dairy herds to stop the ongoing need for treatment of individual animals. Eradication from dairy herds can be achieved with “Blitz therapy”, whereby all infected animals are detected and treated simultaneously^52,53^. However, this approach would need to be combined with good external and internal biosecurity to prevent re-introduction and onward transmission within dairy herds, a scenario that appears to be quite common in Colombia based on the within-farm strain heterogeneity that we observed. When considering treatment of the whole herd to eradicate GBS, susceptibility testing should be undertaken to inform drug selection.

Regardless of the choice of interpretation criteria, the prevalence of apparent phenotypic macrolide and tetracycline resistance was only 10 to 14 percentage points higher in human isolates than in bovine isolates. This contrasts with results reported from New York State in the USA, where macrolide and tetracycline resistance were significantly more prevalent in human isolates than in bovine isolates^26^. The difference may be related to the fact that in the USA, GBS screening in all pregnant women and antimicrobial treatment in all positive cases has been implemented since the 1990s^31^, whereas GBS is not that common on dairy farms due to better implementation of the 5-point plan compared to Colombia. Conversely, in Colombia, GBS screening and treatment of GBS positive pregnant women was recommended by health authorities only in 2013^54^, GBS prevalence in dairy farms is high and antimicrobial treatment of cows is common. The prevalence of *tet*(M) among human isolates was low in comparison with the “global” study conducted by Da Cunha and colleagues^55^. That study did not include a single isolate from South America. In addition, their study preferentially included invasive isolates, specifically ST17, whereas our study focused on carriage isolates, with only a few isolates belonging to ST17. Differences in clinical and geographical origin of the isolates may explain the observed difference in tetracycline resistance and serve as further warning that extrapolation and comparison of study results must be conducted with caution and epidemiological insight.

In GBS, tetracycline resistance is primarily due to ribosomal protection and encoded by *tet*(M) or *tet*(O)^56^. In our study, *tet*(M) was the most common resistance gene in human and bovine isolates. These results contrast to those from the USA, where bovine GBS isolates predominantly carried *tetO* and human GBS isolates predominantly carried *tetM*^26^. This comparison demonstrates the importance of local surveillance of AMR to detect and address regional differences in prevalence and drivers.

In conclusion, the results of this study suggest that on-farm interspecies transmission of GBS between people and cattle is possible, particularly for ST1, which is a strain capable of colonizing different hosts and environments, including the human throat and rectum, the bovine udder and rectum, and the farm environment. Other strains, which are currently mainly found in cattle, such as members of CC61/67 and CC103, could emerge from the bovine reservoir as a commensal or pathogen of humans. In addition, the apparent prevalence of penicillin resistance was surprisingly high in human and bovine isolates. Further investigation of this phenomenon using *in vitro* and *in silico* methods is needed and could lead to modification of standard treatment recommendations as well as greater emphasis on biosecurity within and between dairy herds.

## Materials and Methods

### Bioethics

This study was approved by the bioethics councils for human and animal experimentation of the Universidad de Caldas, Manizales, Colombia (documents CBCS023-15 and 130705B-15), and all research was conducted in accordance with the relevant guidelines and regulations. Written informed consent was obtained from all participants or legal guardians for those younger than 18 years of age. Owners of the farms selected for conducting this work gave their permission to collect bovine and environmental samples.

### Farm selection

This study was conducted between July 2016 and October 2017. To be included in the study, farms had to be located in the province of Caldas, Colombia; to have been GBS positive by bacteriological culture (with species identity of isolates confirmed by PCR) in two out of three bulk tank milk samples collected at weekly intervals one to three months prior the start of this study. In addition, consent had to be given by the owner (for collection of samples from lactating dairy cows) and by at least one milker (for participation in human sampling). Farms were located at 5.3 to 36.1 km from the laboratory, with transport times ranging from 0.2 to 3.5 hours.

### Human sampling

In total, 191 people living or working on 33 farms participated in the study. Most participants were men (n = 139, 73%), and more than a half of the participants were directly involved in the milking process (n = 101, 53%). With one exception, all men lived on the farm. Median age of participants was 36 years (range: 16–59 years).

Throat (n = 191) and rectal (n = 189) swabs were collected by trained nurses. Swabs were collected and transported using Cary Blair medium (BD™, Franklin Lakes, NJ, USA). Swabs were transported to the laboratory of milk quality at Universidad de Caldas and immediately placed in 2 ml of Todd Hewitt Broth (THB) (BBL™, Franklin Lakes, NJ, USA) with 0.01 mg/ml colistin sulphate and 5 µg/ml oxoline acid (COBA™ Streptococcus selective supplement, Oxoid, UK). Samples were incubated for 24 hours at 37 °C.

### Cow sampling

Milk samples were taken from all 2092 lactating cows (11 to 165 per farm; median 56) in the 33 herds. After udder preparation for milking, teat ends were disinfected with 70% ethanol and the first streaks of milk were discarded. Composite samples, i.e. approximately equal volumes of milk from each functional mammary quarter were collected into a 30 mL sterile vial. Samples were immediately transported to the laboratory under refrigeration.

Rectal swabs were collected from 1922 of the cows (2 to 159 samples per farm; median 53) included in milk sampling. Swabs were collected using sterile cotton wool swabs, and immediately placed in 2 ml of THB, with streptococcus selective supplement COBA™ (Oxoid, UK). Inoculated THB were transported to the laboratory under refrigeration. Enrichments were incubated for 24-hour at 37 °C.

### Environmental sampling

After milking, areas in feeders where cows had been eating/licking, as evidenced by wet spots, were rubbed with cotton wool swabs that had been moistened with THB with *Streptococcus* selective supplement COBA™ (Oxoid, UK). From the 33 herds, 128 swabs were collected (2 to 11 per herd). Swabs from the internal walls of 47 drinking water containers were collected in 24 herds (Range: 1 to 4 per herd) using sterile cotton swabs. All swabs (n = 175) were processed as described for bovine rectal swabs.

### GBS identification

Milk samples and enrichments (for swabs) were plated onto a chromogenic agar (Strepto B Chrome ID®, Biomerieux, Marcy l’Etoile. France) and incubated for 24–48 hours at 37 °C. Potential GBS isolates (light pink to dark red colonies) were subcultured onto blood-esculin agar, using one colony per morphotype. Plates were aerobically incubated overnight at 37 °C. Esculin-negative colonies were diluted in 20 µL of distilled water. Ten microliters were used as template for GBS confirmation by PCR^57^, using the primers STRA-AgI (AAGGAAACCTGCCATTTG) and STRA-AgII (TTAACCTAGTTTCTTTAAAACTAGAA), which target the 16S–23S intergenic spacer region, with amplicons of 270 bp^58^. The remaining 10 µL of the colony suspension were subcultured in Todd Hewitt Broth and incubated for 24 h at 37 °C. Overnight cultures were archived at −80 °C with 20% glycerol (v/v). This procedure ensured that species confirmation and subsequent MLST and susceptibility testing were conducted on the same isolate. An isolate is defined here as “a population of microbial cells in pure culture derived from a single colony on an isolation plate and characterized by identification to the species level”, in accordance with European and American guidelines^59^. In accordance with those same guidelines, the term “strain” is reserved for “An isolate or group of isolates exhibiting phenotypic and/or genotypic traits which are distinctive from those of other isolates of the same species”, such as the antimicrobial resistance (AMR) profile or sequence type (ST).

### Multi-locus sequence typing

High-throughput multi-locus sequence typing (HiMLST; Streeklab Haarlem, Haarlem, The Netherlands^60^) was used to characterize a single GBS isolate for each sample. Sequencing of standard MLST loci was conducted using an Illumina MiniSeq™ (Illumina, Inc. California, US) and allelic profiles and STs were assigned using the publicly available on-line *Streptococcus agalactiae* database in PubMLST^61^.

### Antimicrobial resistance profiles

Minimum inhibitory concentrations (MIC) of ampicillin, penicillin, erythromycin and tetracycline were determined by microdilution method, using Sensititre Mastitis Plate™ (Thermo Fisher Scientific. Waltham, USA). Compounds were selected based on their relevance as drug of 1^st^ or 2^nd^ choice for GBS treatment (penicillin, erythromycin) or their common use on Colombian dairy farms (ampicillin, tetracycline). After one to two years of storage, 269 of the stored isolates from farmers and bovines could be recovered for MIC determination. After revival on blood-esculin agar (37 °C, 24 hours) one colony per plate was diluted in ultra-pure water to 0.5 McFarland. Fifty microliters were then added to 5.5 mL of cation-adjusted Mueller Hinton Broth (MHB) and agitated, with 50 µL of MHB subsequently used as inoculum for the commercial plate. After 24 hours of incubation at 37 °C, plates were read using the Sensititre Vizion™ visualizer (Thermo Fisher Scientific. Waltham, USA). A *Streptococcus pneumoniae* strain (ATCC49619) with known MIC was processed every work session. MIC breakpoints have not been defined for bovine GBS. Instead, data were interpreted based on breakpoints for human GBS as set by the Clinical Laboratory Standards Institute^48^ and the European Committee for Antimicrobial Susceptibility Testing^62^ (Supplementary Table S3).

### Resistance genes

DNA was extracted from all GBS isolates used for resistance phenotyping with a Bacteria DNA Preparation Kit™ (Jena Bioscience. Jena, Germany). DNA was used for PCR to detect macrolides resistance genes (*ermA, ermB* and *mefA)*^63^ and tetracycline resistance genes (*tetM, tetO* and *tetK*)^64^. Primers are listed in Supplementary Table S4.

### Data analysis

Statistical analysis was conducted using Stata 15 (StataCorp™, College Station, Texas, USA). Descriptive statistics and frequency tables were generated. Unconditional associations between categorical variables were explored using Pearson χ^2^ or Fisher exact test, as appropriate. For analysis of MLST data, the complete up-to-date MLST database available on 5 June 2018 (https://pubmLst.org/sagalactiae) was used for a comparative electronic analysis based upon related sequence types, eBURST (http://eburst.mLst.net/)^65^, and to create a population snapshot. Closely related STs were assigned to clusters or clonal complexes (CC), using sharing of five of seven alleles to define CCs. Antimicrobial resistance patterns were mapped onto CCs using the goeBURST algorithm in PHYLOViZ (http://www.phyloviz.net/). The association between STs and AMR phenotype or genotype was analyzed for STs with at least 10 isolates.

## Supplementary information

Supplementary Information.

## Data Availability

All data generated or analyzed during this study are included in this published article and its Supplementary Information File. Additional information is available from the corresponding author on reasonable request.
