# Supplementary material for: Potential group B Streptococcus interspecies transmission between cattle and people in Colombian dairy farms
Source: Sci Rep. 2019 Oct 1;9:14025. doi: 10.1038/s41598-019-50225-w (PMC6773701; doi:10.1038/s41598-019-50225-w)
Supplement: Supplementary file 1 — Supplementary Information. [file 41598_2019_50225_MOESM1_ESM.docx]

**Supplementary Information**

**Potential group B *Streptococcus* interspecies transmission between cattle and people on Colombian dairy farms**

Claudia G. Cobo-Ángel^1^; Ana S. Jaramillo-Jaramillo^1^; Monica Palacio-Aguilera^2^; Liliana Jurado-Vargas^2^; Edwin A. Calvo-Villegas^2^; Diego A. Ospina-Loaiza^1^; Juan C. Rodriguez-Lecompte^3^; Javier Sanchez^3^; Ruth Zadoks ^4 5^; Alejandro Ceballos-Márquez^1^

^1^ Research Group in Milk Quality and Veterinary Epidemiology, Faculty of Agricultural Sciences, Universidad de Caldas, Manizales, Colombia

^2^ Faculty of Health Sciences, Universidad de Caldas, Manizales, Colombia

^3^ Atlantic Veterinary College, University of Prince Edward Island, Charlottetown, Prince Edward Island, Canada

^4^ Institute of Biodiversity, Animal Health and Comparative Medicine, College of Medical, Veterinary and Life Sciences, University of Glasgow, Glasgow, UK

^5^ Sydney School of Veterinary Science, University of Sydney, Camden, NSW, Australia

**Supplementary Table S1**. Number of human, bovine, and environmental samples collected and positive for group B *Streptococcus* (GBS) in each farm. ST = sequence types detected among GBS isolates from the specified origin.

| Farm | Human | | | Bovine | | | Environment | | |
| --- | --- | --- | --- | --- | --- | --- | --- | --- | --- |
|  | **Sampled** | **GBS** | **ST** | **Sampled** | **GBS** | **ST** | **Sampled** | **GBS** | **ST** |
| 1 | 7 | 0 |  | 165 | 6 | 356 | 7 | 0 |  |
| 2 | 5 | 4 | 1, 12, 26 | 42 | 11 | 1, 718, 1175 | 5 | 0 |  |
| 3 | 4 | 1 | 1 | 77 | 6 | 248, 314 | 6 | 1 | 1 |
| 4 | 4 | 2 | 1 | 80 | 20 | 1, 718, 1149, 1175 | 4 | 0 |  |
| 5 | 3 | 1 | 26 | 101 | 35 | 1 | 7 | 0 |  |
| 6 | 3 | 4 | 1, 23, 24 | 62 | 8 | 1, 356, 1149 | 6 | 1 | 356 |
| 7 | 3 | 0 |  | 55 | 14 | 1 | 3 | 0 |  |
| 8 | 6 | 1 | 23 | 72 | 9 | 356 | 5 | 0 |  |
| 9 | 9 | 8 | 8, 17, 23, 24, 1175 | 75 | 20 | 356 | 5 | 0 |  |
| 10 | 4 | 1 | 1 | 26 | 6 | 1 | 5 | 0 |  |
| 11 | 4 | 2 | 23 | 25 | 6 | 1, 718 | 3 | 0 |  |
| 12 | 3 | 2 | 55 | 73 | 16 | 1, 248 | 10 | 0 |  |
| 13 | 6 | 0 |  | 88 | 2 | 1, 718 | 8 | 0 |  |
| 14 | 6 | 1 | 1 | 105 | 15 | 718 | 9 | 0 |  |
| 15 | 3 | 0 |  | 37 | 1 | 718 | 4 | 0 |  |
| 16 | 6 | 2 | 24, 1175 | 111 | 0 |  | 8 | 0 |  |
| 17 | 9 | 1 | 1 | 118 | 12 | 1, 356 | 9 | 2 | 356 |
| 18 | 6 | 1 | 130 | 41 | 8 | 1, 248, 356, 718 | 6 | 0 |  |
| 19 | 5 | 1 | 1 | 11 | 0 |  | 2 | 0 |  |
| 20 | 3 | 0 |  | 45 | 3 | 1, 356 | 0 | 0 |  |
| 21 | 12 | 1 | 23 | 105 | 4 | 1, 718 | 9 | 0 |  |
| 22 | 9 | 0 |  | 17 | 0 |  | 5 | 0 |  |
| 23 | 9 | 1 | 248 | 65 | 3 | 356 | 6 | 0 |  |
| 24 | 5 | 2 | 1 | 23 | 3 | 356 | 3 | 0 |  |
| 25 | 11 | 1 | 17 | 130 | 33 | 61, 718, 1149 | 5 | 0 |  |
| 26 | 4 | 0 |  | 43 | 0 |  | 9 | 0 |  |
| 27 | 7 | 5 | 1, 130 | 27 | 20 | 356, 718 | 1 | 0 |  |
| 28 | 11 | 2 | 1, 23 | 80 | 16 | 718, 1149 | 6 | 0 |  |
| 29 | 4 | 0 |  | 56 | 3 | 1, 356, 1175 | 4 | 0 |  |
| 30 | 7 | 4 | 24, 88, 248 | 48 | 8 | 1 | 5 | 0 |  |
| 31 | 2 | 2 | 1, 24 | 19 | 0 |  | 2 | 0 |  |
| 32 | 5 | 8 | 1, 88, 718 | 20 | 6 | 718 | 4 | 0 |  |
| 33 | 6 | 2 | 1, 23 | 50 | 7 | 1 | 4 | 1 | 1 |

**Supplementary Table S2**. Distribution of erythromycin (*erm, mef)* and tetracycline (*tet)* resistance genes by sequence type (ST) and host species. Prevalence of resistance gene expressed as proportion relative to total number of isolates within each host species.

|  | **ST1** | **ST8** | **ST12** | **ST17** | **ST19** | **ST23** | **ST24** | **ST26** | **ST55** | **ST61** | **ST130** | **ST248** | **ST314** | **ST356** | **ST718** | **ST1149** | **ST1175** | **Total**  **n (%)** |
| --- | --- | --- | --- | --- | --- | --- | --- | --- | --- | --- | --- | --- | --- | --- | --- | --- | --- | --- |
| **Human** |  |  |  |  |  |  |  |  |  |  |  |  |  |  |  |  |  |  |
| *erm*A | 1 | 0 | 0 | 0 | 0 | 2 | 1 | 3 | 1 | n/a | 0 | 0 | n/a | n/a | n/a | n/a | 0 | 8 (24) |
| *erm*B | 5 | 0 | 0 | 1 | 0 | 1 | 1 | 1 | 0 | n/a | 2 | 1 | n/a | n/a | n/a | n/a | 1 | 13 (39) |
| *mef*A | 2 | 0 | 0 | 0 | 0 | 0 | 0 | 0 | 0 | n/a | 0 | 0 | n/a | n/a | n/a | n/a | 0 | 2 (6) |
| *tet*(K) | 2 | 0 | 0 | 0 | 0 | 1 | 0 | 0 | 0 | n/a | 0 | 0 | n/a | n/a | n/a | n/a | 0 | 3 (9) |
| *tet*(M) | 3 | 1 | 0 | 0 | 1 | 6 | 2 | 1 | 1 | n/a | 0 | 1 | n/a | n/a | n/a | n/a | 2 | 18 (55) |
| *tet*(O) | 2 | 0 | 0 | 0 | 0 | 1 | 1 | 1 | 1 | n/a | 0 | 0 | n/a | n/a | n/a | n/a | 0 | 6 (18) |
| Total | 11 | 1 | 1 | 1 | 1 | 6 | 3 | 3 | 1 | 0 | 2 | 1 | 0 | 0 | 0 | 0 | 2 | 33 |
| **Bovine** |  |  |  |  |  |  |  |  |  |  |  |  |  |  |  |  |  |  |
| *erm*A | 22 | n/a | n/a | n/a | n/a | n/a | n/a | n/a | n/a | 0 | n/a | 2 | 0 | 7 | 2 | 0 | 0 | 33 (14) |
| *erm*B | 32 | n/a | n/a | n/a | n/a | n/a | n/a | n/a | n/a | 9 | n/a | 3 | 3 | 39 | 36 | 1 | 2 | 125 (53) |
| *mef*A | 0 | n/a | n/a | n/a | n/a | n/a | n/a | n/a | n/a | 0 | n/a | 0 | 0 | 0 | 0 | 0 | 0 | 0 (0) |
| *tet*(K) | 0 | n/a | n/a | n/a | n/a | n/a | n/a | n/a | n/a | 0 | n/a | 0 | 0 | 0 | 1 | 0 | 1 | 2 (1) |
| *tet*(M) | 86 | n/a | n/a | n/a | n/a | n/a | n/a | n/a | n/a | 3 | n/a | 7 | 0 | 14 | 51 | 5 | 8 | 174 (74) |
| *tet*(O) | 6 | n/a | n/a | n/a | n/a | n/a | n/a | n/a | n/a | 0 | n/a | 2 | 4 | 12 | 5 | 0 | 3 | 32 (14) |
| *Total* | 95 | 0 | 0 | 0 | 0 | 0 | 0 | 0 | 0 | 14 | 0 | 9 | 4 | 45 | 52 | 8 | 9 | 236 |

**Supplementary Table S3**. Breakpoints of Minimum Inhibitory Concentrations (MIC) for susceptible, intermediate and resistant classification of GBS isolates from human and bovines according to the Clinical Laboratory Standards Institute (CLSI) and the European Committee on Antimicrobial Susceptibility Testing (EUCAST).

|  | **CLSI breakpoints** | | | **EUCAST breakpoints** | |
| --- | --- | --- | --- | --- | --- |
|  | Susceptible | Intermediate | Resistant | Susceptible | Resistant |
| Penicillin | ≤0.12 | NR | NR | ≤0.25 | > 0.25 |
| Ampicillin | ≤0.25 | NR | NR | NA | NA |
| Erythromycin | ≤0.25 | 0.5 | ≥1 | ≤0.25 | > 0.5 |
| Tetracycline | ≤2 | 4 | ≥8 | ≤1 | > 2 |

NR: no reported breakpoints for the respective category

NA: not applicable, penicillin testing used as indicator for susceptibility and resistance

**Supplementary Table S4**. Primers used for resistance erythromycin and tetracycline gene amplification from group B *Streptococcus*

| Gene | Primer name | Primer sequence | Amplicon size (bp) | Reference |
| --- | --- | --- | --- | --- |
| Tetracycline resistance | | | |  |
| *tet(*K) | *tetK* F | 5’-TCG ATA GGA ACA GCA GTA-3’ | 169 | Ng et al., (2001) |
|  | *tetK R* | 5’-CAG CAG ATC CTA CTC CTT-3’ |  |  |
| *tet*(M) | *tetM* F | 5’-GTG GAC AAA GGT ACA ACG AG-3’ | 406 |  |
|  | *tetM* R | 5’-CGG TAA AGT TCG TCA CAC AC-3’ |  |  |
| *tet*(O) | *tetO* F | 5’-AAC TTA GGC ATT CTG GCT CAC-3’ | 515 |  |
|  | *tetO* R | 5'-TCC CAC TGT TCC ATA TCG TCA-3’ |  |  |
| Macrolide resistance | | | |  |
| *mefA* | *mefA* F | 5-AGTATCATTAATCACTAGTGC-3’ | 328 | Poyart et al., (2003) |
|  | *mefA* R | 5’-TTCTTCTGGTACTAAAAGTGG-3’ |  |  |
| *erm*A | *ermA* F | 5’-AGACACCTCGTCTAACCTTC-3’ | 423 |  |
|  | *ermA* R | 5’-ATACTTTTTGTAGTCCTTCTT-3’ |  |  |
| *ermB* | *ermB* F | 5’-GGTAAAGGGCATTTAACGAC-3’ | 454 |  |
|  | *ermB* R | 5’-CGATATTCTCGATTGACCCA-3’ |  |  |

**Supplementary Figure S1**. Clusters of group B *Streptococcus* (GBS) as defined by sharing of 5 or more of 7 alleles from the multi-locus sequence typing scheme and their association with susceptibility to ampicillin (AMP), penicillin (PEN), erythromycin (ERY) and tetracycline (TET). The size of the circles and their colored segments is proportional to the number of isolates and the numbers in the circles indicate sequence types. Color key: green: susceptible (all compounds); blue: intermediate (for ERY and TET) or non-susceptible (for AMP and PEN); red: resistant (ERY and TET only). Interpretation of minimum inhibitory concentrations based on guidelines from the Clinical and Laboratory Sciences Institute for human GBS.

**
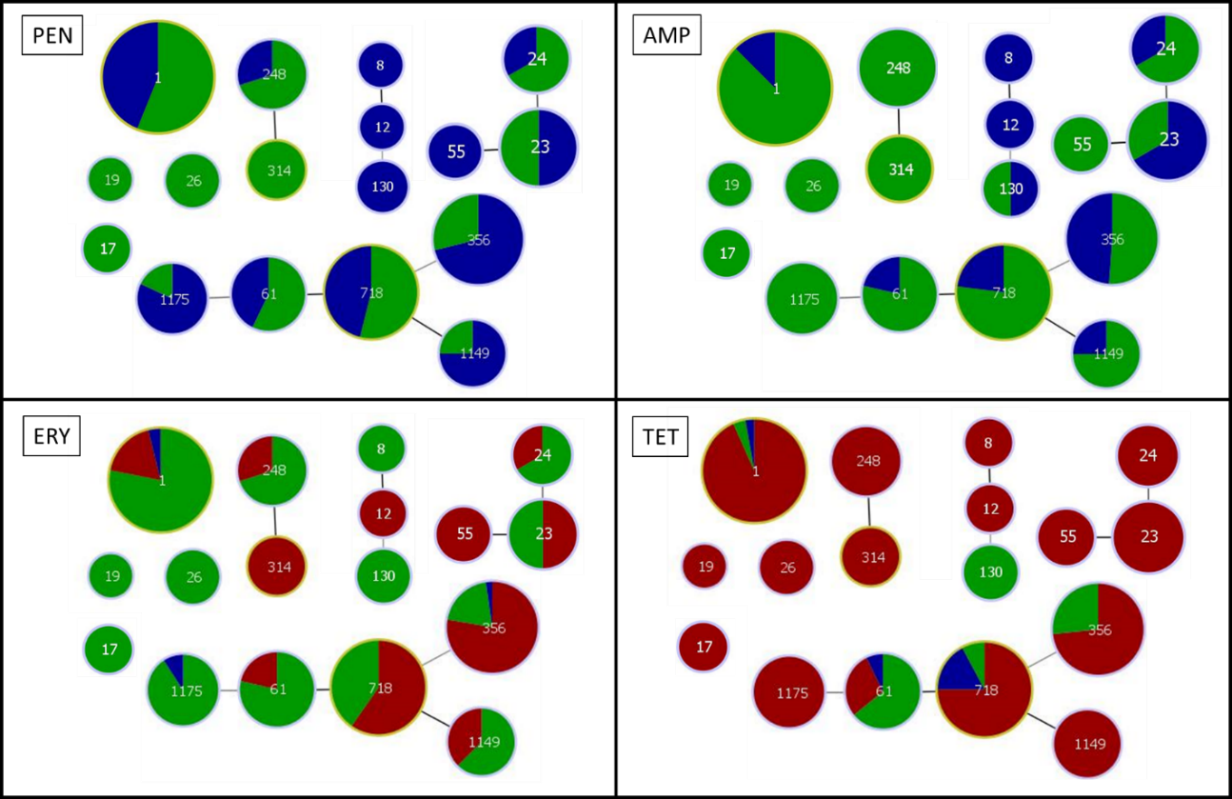
**
